# The Glutamate/GABA‐Glutamine Cycle: Insights, Updates, and Advances

**DOI:** 10.1111/jnc.70029

**Published:** 2025-03-11

**Authors:** Jens V. Andersen

**Affiliations:** ^1^ Department of Drug Design and Pharmacology, Faculty of Health and Medical Sciences University of Copenhagen Copenhagen Denmark

**Keywords:** Alzheimer's disease, astrocytes, lipid metabolism, mitochondrial function, neurodegeneration, neurotransmitter recycling

## Abstract

Synaptic homeostasis of the principal neurotransmitters glutamate and GABA is tightly regulated by an intricate metabolic coupling between neurons and astrocytes known as the glutamate/GABA‐glutamine cycle. In this cycle, astrocytes take up glutamate and GABA from the synapse and convert these neurotransmitters into glutamine. Astrocytic glutamine is subsequently transferred to neurons, serving as the principal precursor for neuronal glutamate and GABA synthesis. The glutamate/GABA‐glutamine cycle integrates multiple cellular processes, including neurotransmitter release, uptake, synthesis, and metabolism. All of these processes are deeply interdependent and closely coupled to cellular energy metabolism. Astrocytes display highly active mitochondrial oxidative metabolism and several unique metabolic features, including glycogen storage and pyruvate carboxylation, which are essential to sustain continuous glutamine release. However, new roles of oligodendrocytes and microglia in neurotransmitter recycling are emerging. Malfunction of the glutamate/GABA‐glutamine cycle can lead to severe synaptic disruptions and may be implicated in several brain diseases. Here, I review central aspects and recent advances of the glutamate/GABA‐glutamine cycle to highlight how the cycle is functionally connected to critical brain functions and metabolism. First, an overview of glutamate, GABA, and glutamine transport is provided in relation to neurotransmitter recycling. Then, central metabolic aspects of the glutamate/GABA‐glutamine cycle are reviewed, with a special emphasis on the critical metabolic roles of glial cells. Finally, I discuss how aberrant neurotransmitter recycling is linked to neurodegeneration and disease, focusing on astrocyte metabolic dysfunction and brain lipid homeostasis as emerging pathological mechanisms. Instead of viewing the glutamate/GABA‐glutamine cycle as individual biochemical processes, a more holistic and integrative approach is needed to advance our understanding of how neurotransmitter recycling modulates brain function in both health and disease.

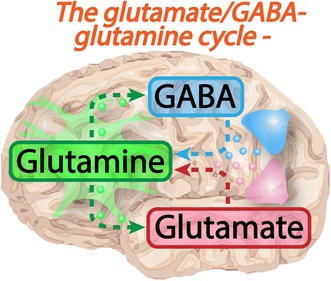

## Graphical abstract

The glutamate/GABA‐glutamine cycle is an intricate metabolic shuttle linking neurotransmission to cellular metabolism. In essence, astrocytes take up synaptic glutamate and GABA and convert these neurotransmitters into glutamine, which is transferred back to the neurons where glutamine serves as the principal precursor for neurotransmitter glutamate and GABA synthesis. The glutamate/GABA‐glutamine cycle is fundamental to brain function in both health and disease, which can only be fully appreciated when viewing the glutamate/GABA‐glutamine cycle as a holistic integrated system.

**Abbreviations**
AAT: aspartate aminotransferase
Ala: alanine
ALAT: alanine aminotransferase
APOE: apolipoprotein E
Asp: aspartate
BCAA: branched‐chain amino acid
BCAT: branched‐chain amino acid aminotransferase
BCKA: branched‐chain α‐keto acid
CaMKIIα: Ca^2+^/calmodulin‐dependent protein kinase II alpha
EAAT: excitatory amino acid transporter
GABA: γ‐aminobutyric acid
GABA‐T: GABA transaminase
GAD: glutamate decarboxylase
GAT: GABA transporter
GDH: glutamate dehydrogenase
GHB: γ‐hydroxybutyrate
GLAST: glutamate aspartate transporter 1
GLT‐1: glutamate transporter‐1
Glu: glutamate
GS: glutamine synthetase
iPSC: induced pluripotent stem cell
LAT: L‐type amino acid transporter
MAS: malate–aspartate shuttle
NMR: nuclear magnetic resonance
OAA: oxaloacetate
PAG: phosphate‐activated glutaminase
PC: pyruvate carboxylase
PDH: pyruvate dehydrogenase
Pyr: pyruvate
ROS: reactive oxygen species
SLC: solute carrier
SNAT: sodium‐coupled neutral amino acid transporter
SSADH: succinic semialdehyde dehydrogenase
TCA: tricarboxylic acid (cycle)
α‐KG: α‐ketoglutarate

## 1 Introduction

**FIGURE 1:**
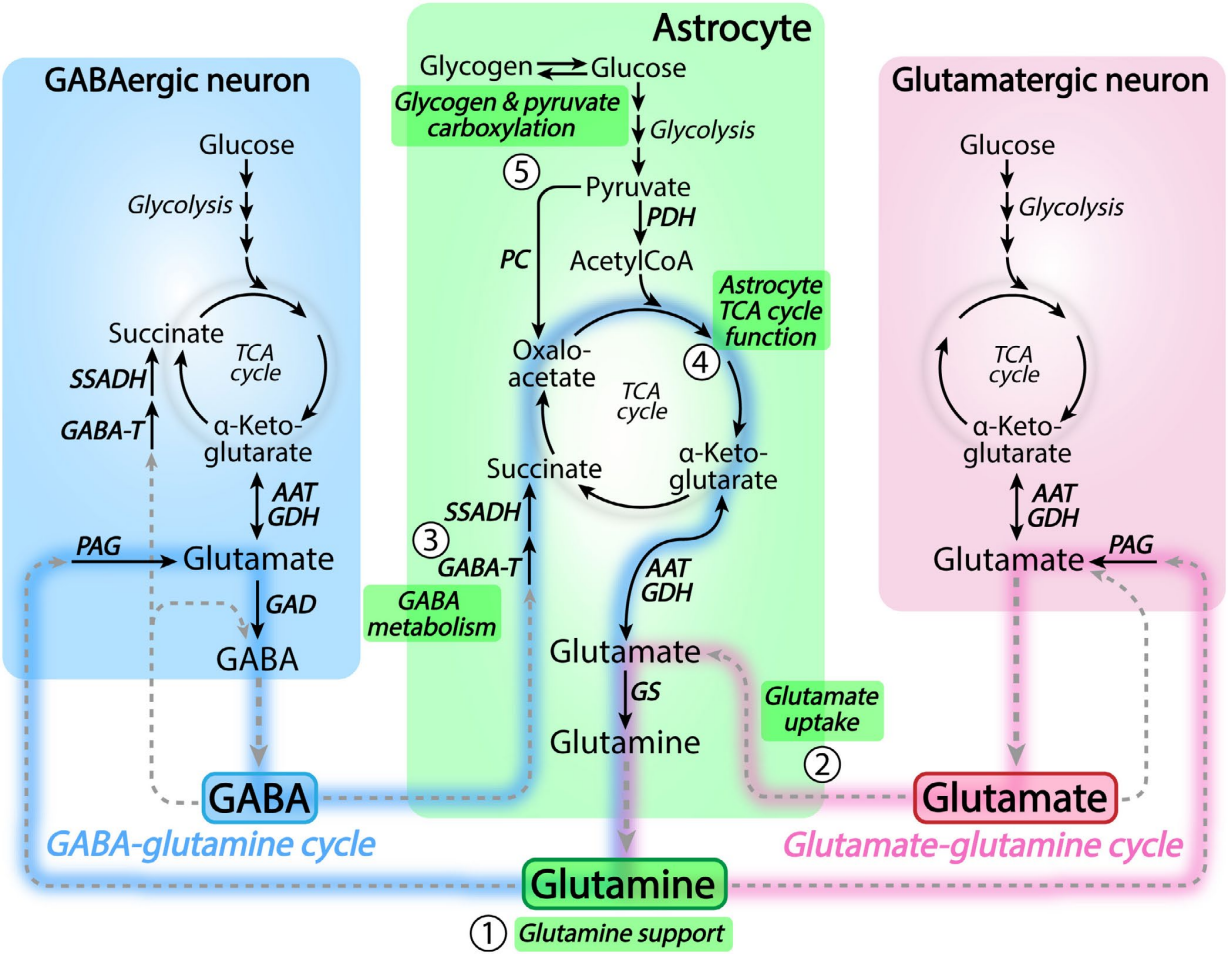
Astrocytes orchestrate the glutamate/GABA‐glutamine cycle. The transcellular recycling of glutamate, GABA, and glutamine is known as the glutamate‐glutamine cycle (right, pink trace) and the GABA‐glutamine cycle (left, blue trace), collectively also called the glutamate/GABA‐glutamine cycle. Astrocytes are at the center of the glutamate/GABA‐glutamine cycle integrating neurotransmitter release, uptake, synthesis and metabolism. Key astrocytic features in relation to the glutamate/GABA‐glutamine cycle are highlighted in green boxes. (1) Critically, astrocytes synthesize and release large quantities of glutamine, which is taken up by neurons, where it serves as the principal precursor for neurotransmitter glutamate and GABA synthesis. (2) The majority of synaptic glutamate is recovered from the synapse by uptake into astrocytes, which is essential to prevent excitotoxic overstimulation. (3) Furthermore, a substantial fraction of synaptic GABA is taken up by astrocytes, where it enters the TCA cycle to support glutamine synthesis. (4) The glutamate/GABA‐glutamine cycle is closely connected to energy metabolism and astrocyte TCA cycle function is essential to sustain neurotransmitter recycling. (5) Finally, astrocytes display unique metabolic features, including glycogen metabolism and anaplerosis through pyruvate carboxylation, being important processes to support the extensive glutamine synthesis and export. See Figure 1 for detailed metabolic reactions. AAT, aspartate aminotransferase; GAD, glutamate decarboxylase; GDH, glutamate dehydrogenase; GABA‐T, GABA transaminase; PAG, phosphate‐activated glutaminase; PC, pyruvate carboxylase; PDH, pyruvate dehydrogenase; SSADH, succinic semialdehyde dehydrogenase.

**FIGURE 2:**
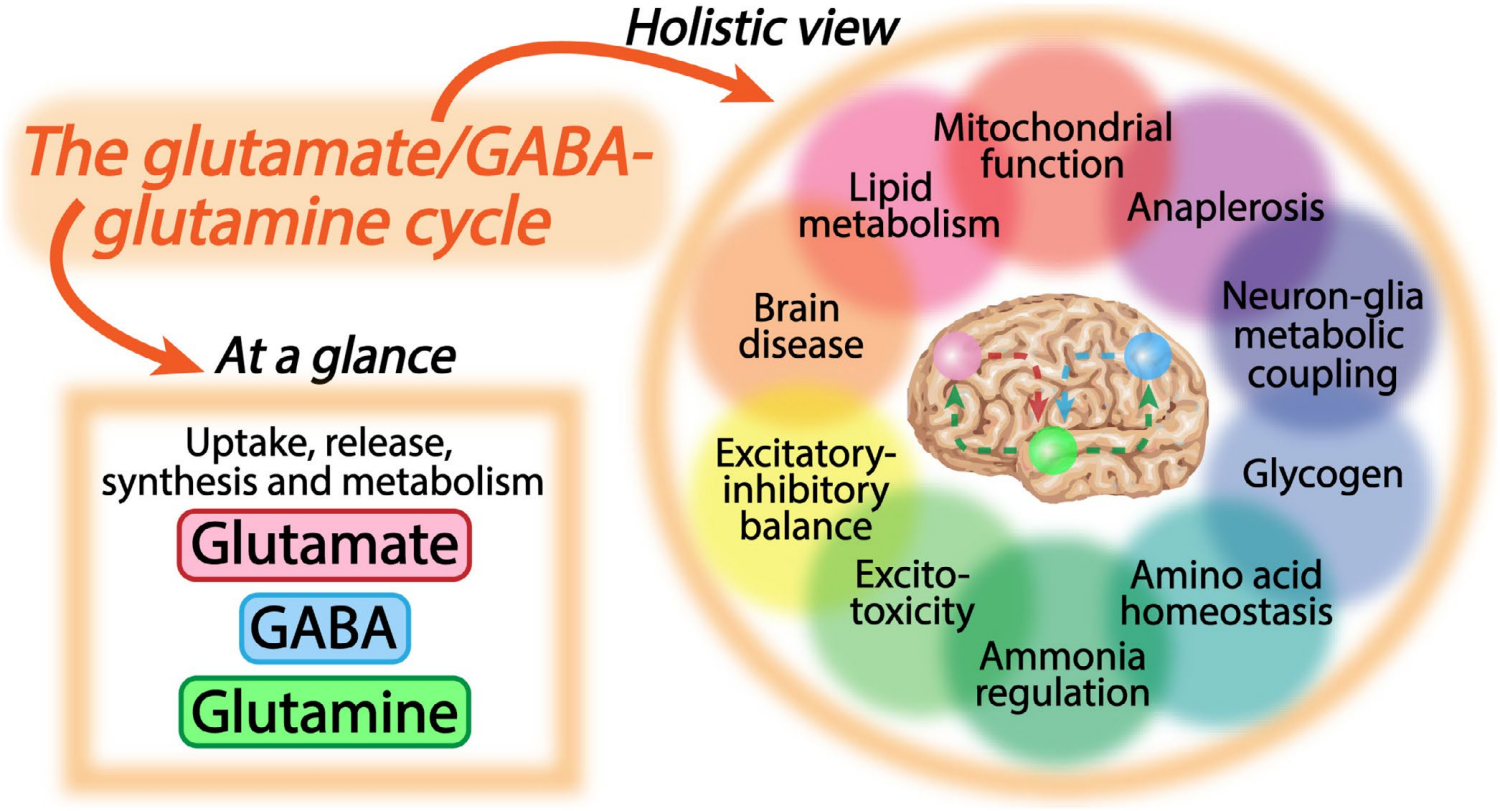
Different views of the glutamate/GABA‐glutamine cycle. At a glance the glutamate/GABA‐glutamine cycle involves an isolated set of reactions relating to release, uptake, synthesis, and metabolism of glutamate, GABA, and glutamine. This is true, however, these reactions are deeply interdependent and so fundamental that they expand across multiple brain functions. Disruption of these processes may profoundly affect brain function, being evident in several brain diseases, and underlines the absolute essentiality of neurotransmitter recycling. A holistic view on the glutamate/GABA‐glutamine cycle as an integrated system is needed to advance our understanding of how neurotransmitter recycling modulates specific brain functions, and vice versa, in both health and disease. Note that the highlighted functional aspects in the holistic view of the glutamate/GABA‐glutamine cycle are not an exhaustive list, but serves as central examples discussed further in this review.

Glutamate and γ‐aminobutyric acid (GABA), being the principal excitatory and inhibitory neurotransmitters, respectively, are extensively recycled between astrocytes and neurons (Schousboe et al. 2013). Astrocytes take up significant fractions of both glutamate and GABA from the synapse (Schousboe, Hertz, et al. 1977; Schousboe, Svenneby, et al. 1977; Schousboe 1981). This astrocytic clearance of synaptic glutamate and GABA will eventually drain the neuronal neurotransmitter pools. To counteract this, astrocytes provide neurons with the non‐neuroactive amino acid glutamine (Albrecht et al. 2007; Andersen and Schousboe 2023b), which is essential to replenish the neuronal glutamate and GABA pools. The cycling of glutamate, GABA, and glutamine between neurons and astrocytes is collectively known as the glutamate/GABA‐glutamine cycle (Figure 1) (Hertz 1979; Bak et al. 2006; Sonnewald and Schousboe 2016). Recycling of glutamate and GABA is critical to maintaining the excitatory‐inhibitory balance and is a prime example of intricate transcellular metabolic coupling within the brain. The glutamate/GABA‐glutamine cycle is a highly complex system integrating many cellular functions, including neurotransmitter release, uptake, synthesis, and metabolism. At a glance, these processes may seem simple, but they are all interdependent and closely coupled to a myriad of brain functions (Figure 2). This makes the glutamate/GABA‐glutamine cycle a fascinating subject to study, but also complicates the mechanistic delineations when the cycle is malfunctioning e.g. during brain disease.

Astrocytes are at the center of the glutamate/GABA‐glutamine cycle (Andersen and Schousboe 2023a) (Figure 1) and actively metabolize both glutamate and GABA to support the synthesis of glutamine. Astrocyte‐derived glutamine is the primary precursor for neuronal glutamate and GABA synthesis (Bradford et al. 1978; Tapia and Gonzalez 1978; Reubi et al. 1978). Glutamine synthesis is a highly active metabolic pathway in astrocytes, and several distinct metabolic features of astrocytes are required to sustain the extensive synthesis and release of glutamine (highlighted in Figure 1). However, other glial cells are emerging as active players in neurotransmitter recycling, which will be emphasized in the following sections. The glutamate/GABA‐glutamine cycle is an open circuit (McKenna 2007) as glutamate, GABA, and glutamine all undergo oxidative metabolism in both neurons and astrocytes (Figure 1). This means that a continuous re‐synthesis of glutamate, GABA, and glutamine is needed to sustain cycle activity, thereby coupling cellular energy metabolism and neurotransmitter recycling. The glutamate/GABA‐glutamine cycle is a major metabolic flux in the brain (Shen et al. 1999; Oz et al. 2004) and the activity of the cycle is directly proportional to cerebral oxidative glucose metabolism (Sibson et al. 1998; Patel et al. 2005).

**FIGURE 3:**
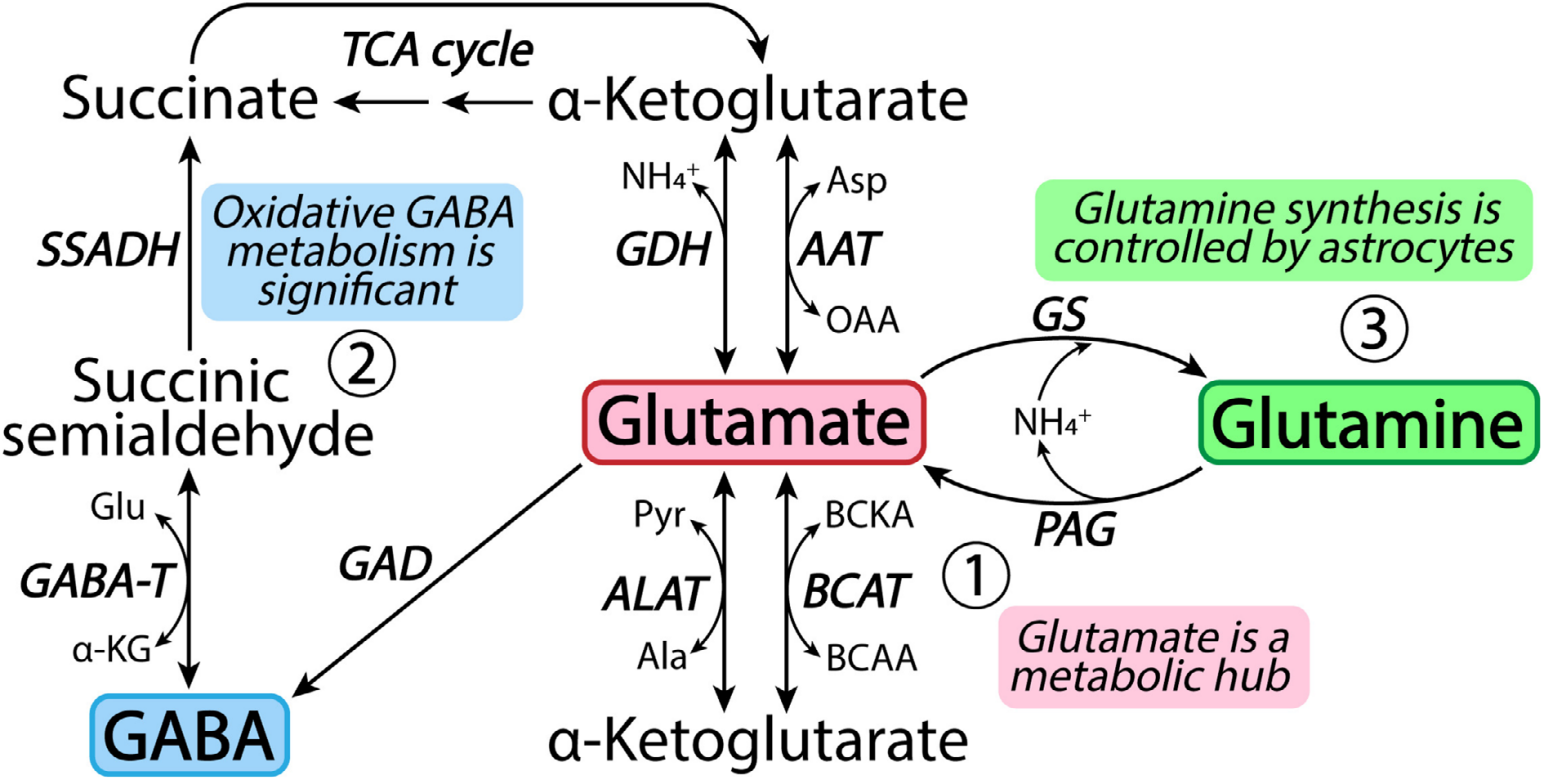
Metabolic roadmap of the glutamate/GABA‐glutamine cycle. Homeostasis of glutamate, GABA and, glutamine is closely coupled through several enzymatic reactions. (1) Glutamate serves as a metabolic hub linking amino acid, neurotransmitter, and energy metabolism through the TCA cycle intermediate α‐ketoglutarate. Glutamate is furthermore the immediate precursor of both GABA and glutamine. The two primary enzymes facilitating glutamate synthesis and metabolism are aspartate aminotransferase (AAT) and glutamate dehydrogenase (GDH), however, alanine aminotransferase (ALAT) and branched‐chain amino acid aminotransferase (BCAT) also catalyze the conversion between glutamate and α‐ketoglutarate. Note that several of these reactions are fully reversible, making glutamate homeostasis highly dynamic. (2) In contrast to glutamate, the irreversible synthesis of GABA is catalyzed by glutamate decarboxylase (GAD). GABA metabolism is facilitated by the successive actions of GABA transaminase (GABA‐T) and succinic semialdehyde dehydrogenase (SSADH) converting GABA into the TCA cycle intermediate succinate for further oxidation. (3) Brain glutamine homeostasis is principally governed by two enzymes: glutamine synthetase (GS) and phosphate‐activated glutaminase (PAG). Glutamine is primarily synthesized in astrocytes by GS activity, which also serves as the primary route of cerebral ammonia (${\mathrm{NH}}_{4}^{+}$) fixation. Conversely, glutamine can be converted back into glutamate by PAG activity, a reaction releasing ammonia. Abbreviations not explained above: α‐KG, α‐ketoglutarate; Ala, alanine; Asp, aspartate; BCAA, branched‐chain amino acid; BCKA, branched‐chain α‐keto acid; Glu, glutamate; OAA, oxaloacetate; Pyr, pyruvate.

Malfunctioning of the glutamate/GABA‐glutamine cycle is a common feature of several brain diseases. This may relate to dysfunctional glutamate and GABA uptake, disrupted glutamine homeostasis, or general cellular metabolic dysfunction. As all parts of the glutamate/GABA‐glutamine cycle are closely coupled to fundamental aspects of brain function, dysfunction of individual processes within the cycle may lead to complex deleterious downstream consequences. The intricacies of neurotransmitter recycling will be highlighted throughout the review and can only be fully appreciated when viewing the glutamate/GABA‐glutamine cycle as an integrated system (Figure 2). The aim of this review is to narrate central elements and recent advances of the glutamate/GABA‐glutamine cycle. First, I outline basic and novel features of cellular glutamate, GABA, and glutamine transport in relation to the cycle. Subsequently, metabolic aspects of the glutamate/GABA‐glutamine cycle are thoroughly reviewed with a special emphasis on astrocyte metabolic function. Finally, advances on neurotransmitter recycling in relation to brain disease are discussed, and a more holistic approach towards the glutamate/GABA‐glutamine cycle is advocated.

**FIGURE 4:**
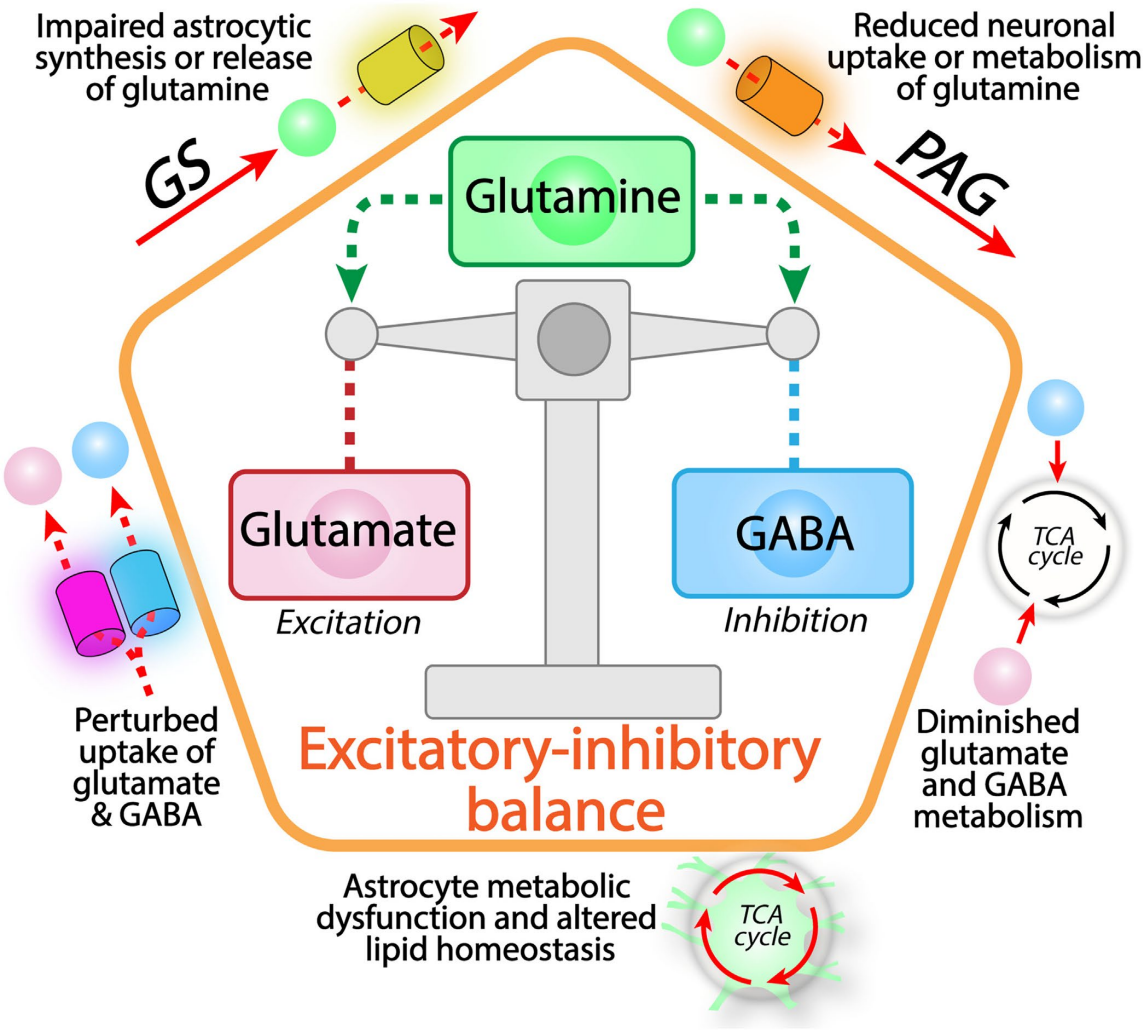
Pathological dysfunction of the glutamate/GABA‐glutamine cycle offsets the signaling balance of the brain. The glutamate/GABA‐glutamine cycle is essential to maintain the delicate cerebral balance of excitatory and inhibitory signaling and thereby overall brain function. As glutamine is the primary precursor of both glutamate and GABA synthesis, dysfunctional glutamine homeostasis can lead to severe disruptions of the glutamate/GABA‐glutamine cycle. This includes impairments of both astrocytic glutamine synthesis and release, but also malfunctioning neuronal uptake and metabolism of glutamine. Additionally, perturbations in cellular uptake or metabolism of synaptic glutamate and GABA, can greatly disturb the excitatory‐inhibitory balance and lead to synaptic dysfunction. Finally, as the glutamate/GABA‐glutamine cycle is intimately coupled to cellular energy metabolism, general impairments of cellular metabolism, including mitochondrial dysfunction, may disrupt neurotransmitter recycling. Astrocyte energy metabolism is particularly crucial to sustain the glutamate/GABA‐glutamine cycle (Figure 1) and dysfunctional astrocyte energetics lead to severe imbalances in brain lipid homeostasis, which is gaining attention in multiple neurological diseases. Note that the depicted dysfunctions are not an exhaustive list, but rather prominent examples of pathological dysfunction of the glutamate/GABA‐glutamine cycle.

## 2 Transport Aspects

### 2.1 Glutamate Transport Is Energy Consuming

Efficient removal of synaptic glutamate is paramount to ensure excitatory signaling with high fidelity and to avoid glutamatergic overstimulation and subsequent excitotoxicity (Danbolt 2001). In the brain, glutamate is cleared from the synapse by several glutamate transporters belonging to the SLC1A family, the three primary being GLT‐1 (*SLC1A2*), GLAST (*SLC1A3*) and EAAT3 (*SLC1A1*) (Amara and Fontana 2002; Martinez‐Lozada and Ortega 2023). The SLC1A system co‐transports one molecule of glutamate across the cell membrane alongside three sodium ions and one proton, with the counter‐transport of one potassium ion (Levy et al. 1998; Zerangue and Kavanaugh 1996; Owe et al. 2006). This large movement of ions is restored by extensive Na^+^/K^+^‐ATPase activity, leading to a great energetic expenditure directly related to synaptic glutamate uptake (Attwell and Laughlin 2001; Yu et al. 2018). GLT‐1, GLAST, and EAAT3 are all expressed throughout the forebrain (Lehre et al. 1995; Rothstein et al. 1994; Schmitt et al. 1997).

GLT‐1 and GLAST expression is highly abundant, and both transporters are particularly enriched in astrocytes (Lehre et al. 1995; Rothstein et al. 1994; Chaudhry et al. 1995). GLAST is the only glutamate transporter displaying completely selective glial expression within the brain (Danbolt et al. 2016) and is particularly enriched in Bergmann glia of the cerebellum (Schmitt et al. 1997). Mice lacking GLAST develop normally but have reduced glutamate uptake in the cerebellum with concurrent impairment of motor coordination (Watase et al. 1998). GLT‐1 is the dominant glutamate transporter of the brain and has been estimated to account for 1% of total brain protein (Lehre and Danbolt 1998), underlining the immense astrocytic capacity for glutamate uptake. However, oligodendrocytes also express several glutamate transporters, including GLT‐1 and GLAST (DeSilva et al. 2009; Pitt et al. 2003). Glutamate uptake by oligodendrocytes is the primary mechanism of glutamate clearance in white matter structures, and dysfunction of this transport system has been implicated in several diseases (Suárez‐Pozos et al. 2020).

Neurons also express glutamate transporters. 5%–10% of all GLT‐1 is located in presynaptic neurons (Chen et al. 2004; Furness et al. 2008; Melone et al. 2009; Zhou, Hassel, et al. 2019). The role of neuronal GLT‐1 remains to be completely established (Rimmele and Rosenberg 2016; Danbolt et al. 2016). Deletion of neuronal GLT‐1 in mice does not alter behavior or general health (Petr et al. 2015), which is in stark contrast to global or astrocytic GLT‐1 deletion leading to severe seizures with early demise (Petr et al. 2015; Rothstein et al. 1996; Tanaka et al. 1997). Despite the mild phenotype, mice lacking neuronal GLT‐1 display disturbances in cerebral glutamate uptake, aspartate homeostasis, cellular energy metabolism, and mitochondrial function (Petr et al. 2015; McNair et al. 2019, 2020; Zhou, Hassel, et al. 2019). In addition, these mice have a heightened vulnerability towards hippocampal excitotoxicity caused by faulty energy metabolism (Rimmele et al. 2021; Li et al. 2024), suggesting a functional role of neuronal glutamate uptake via GLT‐1 in the synaptic microenvironment. Compared to GLT‐1 and GLAST, the expression of EAAT3 is low and is primarily restricted to hippocampal neurons (Conti et al. 1998; Holmseth et al. 2012). Curiously, synaptic glutamate uptake via EAAT3 into neurons is important for GABA synthesis in the hippocampus (Sepkuty et al. 2002). EAAT3 thereby crosslinks glutamatergic and GABAergic signaling by circumventing astrocyte glutamine synthesis and the GABA‐glutamine cycle (Figure 1).

### 2.2 Synaptic GABA Clearance Is Mediated by Both Neurons and Astrocytes

In contrast to glutamate, which is predominantly removed from the synapse by astrocytic uptake, synaptic GABA clearance is divided between presynaptic neurons and astrocytes (Schousboe 1981). The two primary GABA transporters (GATs) are GAT1 (*SLC6A1*) and GAT3 (*SLC6A11*) (Zhou and Danbolt 2013), transporting two sodium ions and one chloride ion alongside one molecule of GABA (Kavanaugh et al. 1992). The initial studies on cellular GAT localization reported a high neuronal expression of GAT1, whereas GAT3 was mainly found in astrocytes (Durkin et al. 1995; Minelli et al. 1995, 1996). However, more recent studies have attributed over 40% of all GAT1 expression in the cerebral cortex to astrocytes (Melone et al. 2015; Fattorini, Melone, and Conti 2020). In addition, GAT1 expression has also been demonstrated in microglia and oligodendrocytes (Fattorini et al. 2017; Fattorini, Catalano, et al. 2020), indicating a diverse cellular interplay at the GABAergic synapse. These observations further suggest that glial GABA uptake through GAT1 may contribute significantly to synaptic GABA clearance, which may have led to underestimations of the glial contribution to synaptic GABA uptake (Andersen et al. 2023). GABA transport and homeostasis differ significantly in the thalamus as GATs are solely located in astrocytes in this brain region (de Biasi et al. 1998). Although GABAergic neurons are highly abundant in the thalamus (Arcelli et al. 1997), thalamic astrocytes synthesize and release GABA to modulate the tonic inhibitory tonus (Kwak et al. 2020). This demonstrates that astrocytes are not passive bystanders, but rather active regulators of thalamic inhibitory transmission.

Deletion of cerebral GAT1 expression in mice leads to chronically elevated extracellular GABA levels offsetting inhibitory signaling (Bragina et al. 2008; Jensen et al. 2003). Furthermore, GAT1 deletion causes altered behavior, but without affecting viability (Chiu et al. 2005; Liu et al. 2007). In contrast to GAT1, no report of a GAT3 knockout model has been presented, presumably because of associated lethality (Zhou and Danbolt 2013). That was until recently, when Ying et al. reported a GAT3 knockout mouse, displaying motor incoordination, imbalance, and impaired learning (Ying et al. 2024). The relatively mild phenotypes of both the GAT1 and GAT3 knockout mice suggest that the two GABA transporters are able to compensate, to some extent, for each other. However, deletion of GAT1 does affect GAT3 expression (Bragina et al. 2008) and vice versa (Ying et al. 2024).

### 2.3 Brain Glutamine Transport Is Governed by Several Mechanisms

Multiple transport systems are capable of facilitating glutamine transport in the brain (Leke and Schousboe 2016); however, the most prominent belong to the SLC38 family (Mackenzie and Erickson 2004), being sodium‐coupled neutral amino acid transporters (SNATs). As the name indicates, the SNATs transport one sodium ion together with one molecule of glutamine, making transport against a cellular concentration gradient possible. In addition, some SNATs, namely SNAT3 (*SLC38A3*) and SNAT5 (*SLC38A5*), are further linked to the antiport of a proton (Chaudhry, Schmitz, et al. 2002). As SNAT3 and SNAT5 are selectively enriched in astrocytes (Boulland et al. 2003; Cubelos et al. 2005), it has been argued that the additional proton‐coupling is needed to facilitate glutamine efflux from these cells (Chaudhry, Schmitz, et al. 2002; Chaudhry et al. 1999; Leke and Schousboe 2016). Knockdown of SNAT3 and SNAT5 in cultured astrocytes also leads to significant intracellular glutamine accumulation mediated by lower glutamine efflux (Zielińska et al. 2016). In line with this, in vivo SNAT3 knockdown reduces extracellular glutamine levels (Hamdani et al. 2021). Global SNAT3 impairment causes brain glutamine accumulation but does not affect plasma glutamine levels (Chan et al. 2016). The elevated cerebral glutamine levels are likely a consequence of impaired astrocyte glutamine release, as both glutamate and GABA levels, primarily located in neurons, were correspondingly decreased (Chan et al. 2016). This observation aligns well with pharmacological inhibition of glutamine transport in guinea pig brain slices, which resulted in glutamine accumulation, whereas glutamate and GABA were depleted (Rae et al. 2003). A large part of astrocyte glutamine export is sodium‐independent, as astrocytes are still capable of releasing glutamine when intracellular sodium stores are exhausted (Deitmer et al. 2003). This was recently suggested to be mediated by the astrocyte hemichannel connexin 43 (Cheung et al. 2022), aiding to sustain glutamatergic transmission via glutamine transfer in the mouse hippocampus.

In neurons, SNAT1 (*SLC38A1*) and SNAT2 (*SLC38A2*) are the primary transporters responsible for glutamine uptake. There is an apparent differential expression of SNAT1 in GABAergic neurons (Solbu et al. 2010; Melone et al. 2004) and SNAT2 in glutamatergic neurons (González‐González et al. 2005; Jenstad et al. 2009), but there may be an overlap of expression (Mackenzie et al. 2003; Melone et al. 2006). Genetic disruption of SNAT1 is associated with disrupted GABA synthesis and transmission caused by reduced glutamine import (Qureshi et al. 2019, 2020), signifying a critical role of SNAT1 in replenishing the neuronal GABA pool.

Several other proteins are capable of facilitating glutamine transport in the brain, including members of the SLC1, SLC6, and SLC7 families (Leke and Schousboe 2016). The SLC7 members LAT1 (*SLC7A5*) and LAT2 (*SLC7A8*) are amino acid exchangers expressed in both neurons and astrocytes (Deitmer et al. 2003; Nagaraja and Brookes 1996; Núñez et al. 2014). As these two transporters are sodium‐independent, it has been argued that they regulate the general cerebral amino acid equilibrium rather than mediating cellular glutamine uptake (Chaudhry, Reimer, et al. 2002; Leke and Schousboe 2016). In contrast, y^+^LAT2 (*SLC7A6*) is a sodium‐dependent amino acid exchanger found in both neurons and astrocytes (Bröer et al. 2000; Bröer and Brookes 2001), albeit with relatively low expression (Deitmer et al. 2003). There are still significant gaps in our knowledge of cerebral glutamine transport, including identifying yet unknown transport mechanisms and understanding how the release and uptake of glutamine are coupled to cellular metabolism (Andersen and Schousboe 2023b).

## 3 Metabolic Aspects

### 3.1 Glutamate Links Energy Metabolism to the Glutamate/GABA‐Glutamine Cycle

Glutamate acts as a central metabolic hub linking the glutamate/GABA‐glutamine cycle to energy metabolism through the TCA cycle intermediate α‐ketoglutarate (Figure 3). The synthesis and metabolism of glutamate can be catalyzed by multiple enzymes, being highly dynamic processes (Schousboe et al. 2014). The two primary enzymes facilitating cerebral glutamate metabolism are glutamate dehydrogenase (GDH) and aspartate aminotransferase (AAT) (McKenna et al. 2016). Both neurons and astrocytes are able to oxidize the carbon skeleton of glutamate in the TCA cycle (Westergaard et al. 1995; McKenna et al. 1996). However, as astrocytes are the primary compartment of synaptic glutamate uptake, they are also the main metabolizers of glutamate (Schousboe, Svenneby, et al. 1977; Schousboe 1981; Danbolt 2001). Much of the glutamate recovered from the synapse by astrocytes is converted directly into glutamine (and takes part in the glutamate/glutamine cycle, Figure 1), but a large fraction is also oxidatively metabolized in these cells. The rate of astrocyte glutamate oxidation is highly concentration‐dependent. At low glutamate concentrations, most glutamate is converted into glutamine, whereas elevated extracellular glutamate levels lead to extensive oxidative metabolism of glutamate in astrocytes (McKenna et al. 1996).

As mentioned above, synaptic glutamate clearance is an energy‐demanding process (Attwell and Laughlin 2001; Yu et al. 2018). Indeed, impairment of astrocyte energy metabolism leads to inadequate glutamate uptake capacity (Swanson et al. 1995; Voloboueva et al. 2007; Di Monte et al. 1999). This may be caused by insufficient ATP generation, making the astrocytes unable to cover the energetic cost of glutamate uptake, or by intracellular build‐up of glutamate due to hampered oxidation. GDH is particularly enriched in astrocytes (Lovatt et al. 2007; Zaganas et al. 2001) and astrocytic deletion of GDH reduces ATP generation (Karaca et al. 2015), illustrating that GDH‐mediated glutamate oxidation is able to support the energetic cost of glutamate uptake (McKenna 2013). Inhibition of GDH impairs astrocyte glutamate uptake capacity (Bauer et al. 2012) and GDH‐deficient astrocytes direct glutamate towards glutamine synthesis rather than oxidation in the TCA cycle (Frigerio et al. 2012; Karaca et al. 2015; Skytt et al. 2012). In mouse astrocytes, elevated AAT activity may compensate in the absence of GDH to reduce the intracellular glutamate levels during high exogenous glutamate concentrations (Skytt et al. 2012; Nissen et al. 2015). This notion is interesting as the mouse brain displays greater AAT expression (Sjöstedt et al. 2020; Bakken et al. 2021) and larger capacity for aspartate generation from exogenous glutamate (Westi et al. 2022) when compared to the human brain. Instead, the human brain expresses an additional isoform of GDH (GDH2), which is not found in rodents (Zhang et al. 2016; Spanaki et al. 2010). Inducing GDH2 expression in mice increases the capacity for astrocytic glutamate oxidation (Nissen et al. 2017). Astrocyte GDH may thereby act as a recruitable metabolic safeguard, ensuring high astrocytic glutamate oxidation during peak concentrations (McKenna 2013). This, in turn, allows sustained astrocyte glutamate uptake, protecting against harmful excitotoxic events.

Finally, glutamate is able to outcompete several other energy substrates, including glucose, lactate, and ketones in astrocytes (McKenna 2012), stressing that glutamate oxidation is a high metabolic priority. Although astrocytes take up and metabolize the majority of synaptic glutamate, neuronal glutamate metabolism must not be neglected. As pointed out above, some neurons express high‐affinity glutamate transporters, and deletion of these neuronal transporters disrupts energy metabolism and mitochondrial function (McNair et al. 2019, 2020). In addition, GDH inhibition also limits synaptic glutamate uptake (Whitelaw and Robinson 2013), whereas genetic deletion of GDH leads to impaired glutamine metabolism in cultured neurons (Hohnholt et al. 2018) and lower glutamate oxidation in isolated synaptosomes (Andersen, Markussen, et al. 2021). Global brain deletion of GDH in mice disrupts both excitatory and inhibitory signaling, exerting profound effects on memory and behavior, which are aggravated by external stress (Lander et al. 2019, 2020; Asraf et al. 2023). In summary, cellular metabolism of glutamate in both astrocytes and neurons is a key component in sustaining neurotransmitter recycling and overall brain function.

### 3.2 GABA Metabolism Is Essential for Brain Function

In contrast to glutamate, the synthesis and metabolism of GABA are catalyzed by three irreversible enzymatic reactions (Andersen et al. 2023). GABA synthesis is facilitated by glutamate decarboxylase (GAD), whereas GABA metabolism is mediated by GABA‐transaminase (GABA‐T) and succinic semialdehyde dehydrogenase (SSADH) (Figure 3). The successive actions of GABA‐T and SSADH convert the carbon skeleton of GABA into the TCA cycle intermediate succinate. In brain slices, GABA is oxidized and released as CO_2_ (Balázs et al. 1970), but when compared to other fuels, GABA is a poor substrate to support brain energy metabolism (Ravasz et al. 2017; Cunningham et al. 1980). However, enzymatic deficits of both GABA‐T and SSADH can lead to severe brain pathologies (Koenig et al. 2017; Malaspina et al. 2016), underlining that brain GABA metabolism is critical for brain health. Both neurons and astrocytes express the metabolic machinery for GABA metabolism, but functionally, astrocytes are the primary compartment of GABA oxidation (Schousboe, Hertz, et al. 1977; Bardakdjian et al. 1979). GABA is able to compete with glutamate for oxidation in astrocytes (McKenna and Sonnewald 2005) and is extensively metabolized to support the synthesis of glutamine (Andersen et al. 2020), which may subsequently aid in sustaining the neuronal GABA pool as part of the GABA‐glutamine cycle (Figure 1). The close metabolic link between GABA and glutamine is emphasized by the severe depletion of brain glutamine when GABA‐T is pharmacologically inhibited (Paulsen and Fonnum 1988; Pierard et al. 1999). Furthermore, a selective reduction in brain glutamine is also observed during SSADH deficiency (Gibson et al. 2002; Kirby et al. 2020), which is accompanied by severe perturbations of astrocyte metabolism and function (Andersen et al. 2024).

Although astrocytes exhibit highly active GABA metabolism, neuronal GABA metabolism should not be disregarded. Both GABAergic and glutamatergic neurons express GABA‐T and SSADH (Bakken et al. 2021), and display active GABA metabolism (Gram et al. 1988). The presence of these enzymes in excitatory neurons may suggest that GABA metabolism is not limited to the inhibitory synapse (i.e., inhibitory neurons and associated astrocytes as illustrated in Figure 1), or that GABA‐T and SSADH could serve alternative cellular functions. The latter notion is supported by studies demonstrating that deletion of SSADH upregulates the expression of mitochondrial proteins (Andersen et al. 2024), which leads to elevated mitochondrial function in cultured excitatory neurons (Afshar‐Saber et al. 2024). This may be explained by the observation that SSADH is crucial for mitophagy (Lakhani et al. 2014), a cellular process disposing of damaged mitochondria (Picca et al. 2023), illustrating an important function of SSADH seemingly unrelated to GABA metabolism.

The intermediate product of oxidative GABA metabolism, succinic semialdehyde (Figure 3), is rapidly converted to succinate facilitated by mitochondrial metabolon formation of GABA‐T and SSADH, and by the very high substrate affinity of SSADH (Cash et al. 1978; Hearl and Churchich 1984). However, a small fraction of succinic semialdehyde is also converted into the metabolite γ‐hydroxybutyrate (GHB) (Snead 3rd and Gibson 2005). Endogenous cerebral levels of GHB are in the range of 2–4 μM in the rodent, whereas 5–20 μM of GHB is present in the human brain (Snead 3rd and Morley 1981). GHB has been suggested to be a neurotransmitter (Maitre 1997), but this claim remains controversial, as several aspects, including synaptic release and transport of endogenous GHB, remain to be fully established (Bay et al. 2014). The elusive high‐affinity binding site of GHB in the brain was recently demonstrated to be the Ca^2+^/calmodulin‐dependent protein kinase II alpha (CaMKIIα) (Leurs et al. 2021), which is an abundant postsynaptic enzyme essential for long‐term potentiation and learning (Yasuda et al. 2022). This may suggest that GHB, and thereby GABA metabolism, is involved in the regulation of synaptic plasticity, yet the neurobiological functions of endogenous GHB remain to be elucidated.

### 3.3 Glutamine Homeostasis Is Under Glial Control

Glutamine is a central and highly abundant amino acid in the brain, which is principally synthesized in astrocytes (Andersen and Schousboe 2023b). The seminal discovery of restricted glutamine synthetase (GS) expression in glial cells (Martinez‐Hernandez et al. 1977), more specifically in astrocytes (Norenberg and Martinez‐Hernandez 1979), was a major breakthrough in understanding the complex metabolic compartmentation of the brain (Schousboe 2012). GS is found throughout the brain (Norenberg 1979) and catalyzes the conversion of glutamate and ammonia into glutamine (Figure 3). Glutamine synthesis is essential for brain function. Astrocyte deletion of GS leads to early neonatal mortality (He et al. 2010), which is also observed in congenital human GS deficiency (Häberle et al. 2005). Pharmacological GS inhibition is associated with neuronal depletion of glutamate and GABA (Laake et al. 1995; Fonnum and Paulsen 1990; Andersen, McNair, et al. 2017), which correspondingly disrupts both excitatory and inhibitory neurotransmission (Tani et al. 2014; Ortinski et al. 2010; Liang et al. 2006). These notions emphasize the obligatory role of glutamine in sustaining neuronal transmission through the glutamate/GABA–glutamine cycle (Figure 1).

Glutamine synthesis is furthermore the primary pathway for cerebral ammonia fixation (Felipo and Butterworth 2002). This aspect is critical as elevated cerebral levels of ammonia are neurotoxic and reduced GS function may thereby aggravate the deleterious effects of hyperammonemia (see discussion below). Glutamine synthesis is furthermore intimately linked to the energy metabolism of astrocytes. Not surprisingly, disruption of astrocytic TCA cycle function leads to severe reductions in glutamine synthesis capacity (Fonnum et al. 1997; Swanson and Graham 1994), whereas GS inhibition causes a build‐up of carbon within the astrocyte TCA cycle (Andersen, McNair, Schousboe, and Waagepetersen 2017), signifying that glutamine synthesis is a major metabolic flux in astrocytes.

The immunohistochemical studies showing astrocyte‐specific GS expression (Martinez‐Hernandez et al. 1977; Norenberg and Martinez‐Hernandez 1979) were first challenged by Cammer (1990) demonstrating oligodendrocyte GS expression in the spinal cord, which has been confirmed by several other studies (D'Amelio et al. 1990; Tansey et al. 1991; Xin et al. 2019; Ben Haim et al. 2021). Functionally, deletion of oligodendrocyte GS in mice leads to reduced glutamine levels and disrupted glutamatergic signaling in the midbrain, but does not affect longevity (Xin et al. 2019; Ben Haim et al. 2021). The expression of GS in oligodendrocytes aligns well with reports of pyruvate carboxylase (PC) expression and activity in the same cell type (Amaral, Hadera, et al. 2016; Murin et al. 2009). PC ensures sufficient anaplerosis to sustain glutamine synthesis (Figure 1) (discussed in detail below). Additionally, reports of SNAT expression in oligodendrocytes (Marques et al. 2018; Dennis et al. 2024), alongside the functional consequences of oligodendrocyte GS deletion (Xin et al. 2019; Ben Haim et al. 2021), suggest that these cells play an active role in supplying axons with glutamine in white matter structures (Amaral, Tavares, et al. 2016).

Apart from serving as the principal precursor of neuronal glutamate and GABA synthesis, glutamine is also utilized as a substrate to support oxidative metabolism. Glutamine is first converted into glutamate via phosphate‐activated glutaminase (PAG) and may subsequently be transformed into α‐ketoglutarate to support oxidative metabolism (Figure 3). The carbon skeleton of glutamine readily enters the TCA cycle in acute brain slices (El Hage et al. 2011; Andersen, Christensen, Aldana, et al. 2017; Andersen, Christensen, Nissen, et al. 2017), cultured neurons and astrocytes (Westergaard et al. 1995; Waagepetersen et al. 2001, 2005) and isolated mitochondria (Bak et al. 2008). It should be noted that glutamate derived from glutamine may also undergo transamination reactions (Figure 3), which could lead to overestimations of glutamine oxidation when only assessing metabolic connections between amino acids. Furthermore, artificially low in vitro glucose concentrations may cause excessive cellular oxidation of glutamine. Enzymatic deficiency of PAG leads to cerebral glutamine accumulation, which translates into dysfunctional excitatory signaling and associated encephalopathies (Rumping et al. 2019; Gaisler‐Salomon et al. 2009; van Kuilenburg et al. 2019; Masson et al. 2006). PAG expression and activity are highest in glutamatergic and GABAergic neurons (Laake et al. 1999; Kvamme et al. 2000) and glutamine readily supports neuronal oxidative metabolism (Hohnholt et al. 2018). However, astrocytes also display active glutamine metabolism (Cardona et al. 2015), which may provide metabolic flexibility. Yet the functional roles of astrocyte PAG activity remain to be fully established. Finally, glutamine has also proved to be an important substrate to sustain microglial functions when glucose availability is limited (Bernier et al. 2020).

### 3.4 Astrocyte Pyruvate Carboxylation and Glycogen Are Essential for Glutamine Synthesis

Astrocytes display special metabolic features needed to sustain the glutamate/GABA‐glutamine cycle (Figure 1) (Andersen and Schousboe 2023a). As outlined above, astrocyte glutamine is the primary precursor for neuronal glutamate and GABA synthesis. However, large fractions of glutamate, GABA, and glutamine are lost due to oxidative metabolism in both neurons and astrocytes. This means that astrocytes must hold a large capacity for *de novo* glutamine synthesis. Glutamine is derived from the TCA cycle intermediate α‐ketoglutarate, through glutamate (Figure 3), thereby linking glutamine synthesis to astrocyte energy metabolism. Extensive glutamine synthesis will deplete the astrocytic TCA cycle of α‐ketoglutarate, which may negatively affect TCA cycle function, mitochondrial respiration, and energy production. To counteract the loss of TCA cycle intermediates, a sufficient anaplerotic capacity is needed. Anaplerosis describes metabolic reactions that are capable of replenishing the pools of metabolic intermediates in the TCA cycle (Sonnewald 2014; Brekke et al. 2016; Oz et al. 2012).

Several anaplerotic enzymes are present in the brain, but the quantitatively most significant is PC (Patel 1974). This critical enzyme catalyzes the conversion of pyruvate, under the fixation of bicarbonate (${\mathrm{HCO}}_{3}^{-}$), into the TCA cycle intermediate oxaloacetate (Figure 1), and is selectively expressed in astrocytes (Cesar and Hamprecht 1995; Schousboe et al. 2019). The restricted expression of PC in astrocytes was first demonstrated in primary cultures (Yu et al. 1983) and subsequently in isolated cell fractions (Shank et al. 1985). As mentioned previously, some PC activity may be present in oligodendrocytes (Murin et al. 2009; Amaral, Hadera, et al. 2016), and more controversially, maybe even in neurons (Hassel 2001), but the quantitative importance of this anaplerotic pathway in other cell types remains to be established. Astrocyte PC activity correlates closely with brain activity (Oz et al. 2004) and the metabolic rate of astrocytes (Voss et al. 2020), suggesting that astrocytes are capable of elevating their anaplerotic rate to sustain glutamine synthesis, and hence the glutamate/GABA‐glutamine cycle, during increased neurotransmission. The flux through PC is significant and has been estimated to account for 10%–20% of the total cerebral glucose oxidation (Oz et al. 2004; Duarte and Gruetter 2013; McNair et al. 2022). Deficiency of PC results in low cerebral glutamine levels (Perry et al. 1985), whereas PC activity increases during elevated brain ammonia levels in order to sustain ammonia fixation through astrocyte glutamine synthesis (Figure 3) (Zwingmann et al. 2003). These notions underscore that sufficient PC activity is critical to sustain the extensive synthesis of glutamine in astrocytes (Oz et al. 2012; Gamberino et al. 1997).

Astrocytes are also the primary cellular compartment of cerebral glycogen (Figure 1), being a polymer of glucose units (Obel et al. 2012). Glycogen distribution in the brain is highly heterogeneous, but glycogen is present in most gray matter areas and is particularly abundant in hippocampal structures (Oe et al. 2019; Hirase et al. 2019). Although neurons contain some metabolically active glycogen (Saez et al. 2014), the majority is present in astrocytes, more specifically in astrocyte processes surrounding synapses (Oe et al. 2016). The functional roles of brain glycogen are plentiful and diverse (Markussen et al. 2024). During periods of low glucose availability or high neuronal activity, astrocyte glycogen serves as a local energy reserve, providing fuel to sustain signaling (Brown and Ransom 2007; Wender et al. 2000; Brown et al. 2005). However, glycogen is not only an emergency fuel, but is also continuously synthesized and degraded during normal brain activity (Dienel et al. 2007; DiNuzzo et al. 2019). Astrocytic glycogen metabolism was recently demonstrated to be involved in spinal cord pain sensation (Marty‐Lombardi et al. 2024). Additionally, brain glycogen contains up to 25% glucosamine (Sun et al. 2021), which serves important roles in posttranslational protein glycosylation.

Glycogen is also an essential precursor for astrocyte glutamine synthesis. Blocking the metabolism of glycogen depletes brain glutamine and concomitantly reduces cerebral glutamate levels (Gibbs et al. 2007). In addition, inhibition of glycogen synthesis and metabolism strongly impairs memory formation (Gibbs et al. 2006; Suzuki et al. 2011; Duran et al. 2013), which can be counteracted by exogenous glutamine supplementation (Gibbs et al. 2006, 2007). These notions align well with the observation that pharmacological inhibition of glutamine synthesis leads to cognitive deficits (Gibbs et al. 1996; Son et al. 2019) and extensive glycogen granule accumulation (Phelps 1975; Swanson et al. 1989). Collectively, these studies demonstrate that glycogen serves as a major precursor for glutamine, being critical in sustaining neuronal signaling and learning. Intriguingly, exogenous lactate is also able to rescue the memory deficits induced by inhibition of glycogen metabolism (Gibbs et al. 2007; Suzuki et al. 2011). Indeed, astrocyte glycogen can be utilized to support lactate production (Dringen et al. 1993) and the protective effects of lactate have been attributed to enhancing neuronal metabolism and long‐term potentiation (Suzuki et al. 2011; Alberini et al. 2018). However, since astrocytes are also able to utilize exogenous lactate for glutamine production (Gandhi et al. 2009; Gallagher et al. 2009), the beneficial effects of lactate supplementation could also, in part, be mediated by enhanced astrocyte glutamine synthesis. More detailed studies are needed to map the exact metabolic relationship between glycogen, glutamine, and lactate. The studies above highlight that both astrocyte PC activity and glycogen are essential to maintain adequate glutamine synthesis. The restricted astrocytic expression of GS, PC, and glycogen signifies that astrocytes are the principal metabolic regulators of neuronal glutamate and GABA synthesis (Schousboe et al. 2013).

### 3.5 Astrocyte Mitochondrial Function Is Critical for Neurotransmitter Recycling

Due to the immense energy costs of restoring ion gradients following synaptic transmission, neurons have been crowned as the primary energy consumers of the brain. It is estimated that the great neuronal energy demand only leaves 10%–20% of the brain's energy expenditure to astrocytes (Attwell and Laughlin 2001; Harris et al. 2012; Yu et al. 2018), which has prompted the idea that astrocytes are metabolically inert cells when compared to neurons. However, as pointed out by Hertz decades ago (Hertz 1979), astrocytes must possess a significant oxidative metabolic capacity in order to facilitate high‐affinity uptake and subsequent metabolism of glutamate and GABA. Astrocytes do indeed display a high rate of oxidative metabolism (Hertz et al. 2007) and recent reevaluations of astrocyte energetics suggest that these cells are much more energy demanding than previously assumed.

Astrocytes are central in buffering the extracellular rise in potassium following glutamatergic transmission facilitated by extensive astrocyte Na^+^/K^+^‐ATPase activity (MacAulay 2020). Taking this substantial astrocytic Na^+^/K^+^‐ATPase activity into account in the cerebral energy budget, astrocytes may in fact be as energetically expensive as neurons (Barros 2022). In addition, when metabolic in vivo studies are adjusted to the volume fraction of astrocytes, astrocytic glucose oxidation may even exceed that of neurons (Dienel and Rothman 2020). Nuclear magnetic resonance (NMR) studies have concluded that 20%–30% of all cerebral TCA cycle activity occurs in astrocytes (Oz et al. 2004; Sonnay et al. 2018; Blüml et al. 2002), yet astrocytes are often described as primarily glycolytic cells with low mitochondrial activity. This claim is partly supported by the natural inhibition of pyruvate dehydrogenase (PDH) activity in astrocytes (Halim et al. 2010) and inefficient astrocyte mitochondrial supercomplex formation (Lopez‐Fabuel et al. 2016). Astrocytes are also less sensitive to deprivation of oxygen and glucose when compared to neurons (Almeida et al. 2002) and can survive severe mitochondrial damage (Supplie et al. 2017). These observations have fostered the idea that astrocyte energy requirements can be sustained by glycolytic activity with little need for mitochondrial oxidation (Belanger et al. 2011; Magistretti and Allaman 2015). However, recent electron microscopy mappings have revealed dense mitochondrial networks in astrocytes (Agarwal et al. 2017; Aten et al. 2022). This astrocytic network of mitochondria is particularly important during development where it is critical for synaptogenesis (Zehnder et al. 2021). Furthermore, the total mitochondrial content relative to cell volume is similar between neurons and astrocytes (Calì et al. 2019; Aten et al. 2022) and mitochondrial occupancy within fine astrocytic processes is comparable to that of excitatory terminals (Agarwal et al. 2017). Such widespread and abundant mitochondrial distribution in astrocytes is not compatible with low mitochondrial function.

An often‐overlooked aspect, when considering mitochondrial function in neurons and astrocytes, is that these cell types may not utilize the same energy substrate to support oxidative metabolism. In particular, astrocytes can utilize fatty acids as energy substrates, which is not the case for neurons (Fecher et al. 2019; Edmond et al. 1987; Eraso‐Pichot et al. 2018; Andersen, Westi, et al. 2021; Ameen et al. 2024). Selective impairment of astrocyte mitochondrial function greatly disrupts brain lipid homeostasis (Mi et al. 2023). Whereas disruption of long‐chain fatty acid oxidation in astrocytes impairs cognitive performance and reorganizes mitochondrial supercomplex formation (Morant‐Ferrando et al. 2023). The metabolic coupling of astrocytes and neurons through lipid exchange is gaining scientific momentum and may play prominent roles in several diseases (discussed further below).

In relation to the glutamate/GABA‐glutamine cycle, astrocyte glutamate uptake requires large amounts of energy. As outlined previously, malfunction of the astrocytic TCA cycle directly impairs glutamate uptake capacity, leading to neuronal excitotoxicity (Voloboueva et al. 2007; Swanson et al. 1995; Di Monte et al. 1999). Astrocyte mitochondria are also recruited by active glutamate transporters in order to sustain synaptic glutamate clearance (Genda et al. 2011; Jackson and Robinson 2018; Stephen et al. 2015). As glutamine is derived directly from the astrocytic TCA cycle (Figure 1) sufficient TCA cycle activity is also a prerequisite for sustained astrocyte glutamine synthesis (Fonnum et al. 1997; Swanson and Graham 1994). Finally, as described above, astrocytes extensively metabolize both glutamate and GABA in the TCA cycle, which is critical to maintain the homeostasis of these transmitters. In summary, astrocytes display highly active oxidative metabolism, and sufficient mitochondrial function of astrocytes is not only important to sustain the glutamate/GABA‐glutamine cycle but is required for overall brain function and health.

## 4 Disease Aspects

The glutamate/GABA‐glutamine cycle is essential for synaptic function and balancing excitatory and inhibitory signaling. Multiple aspects of the glutamate/GABA‐glutamine cycle can malfunction, which may lead to serious downstream consequences (Figure 4). Such dysregulation may entail disrupted glutamine homeostasis, perturbed uptake or metabolism of glutamate and GABA, or a more generalized metabolic dysfunction. As the glutamate/GABA‐glutamine cycle is intimately coupled to astrocyte energy metabolism (Figure 1), the metabolic dysfunction of these cells may in particular impair neurotransmitter recycling.

### 4.1 Disrupted Glutamine Homeostasis May Lead to Serious Cellular Dysfunction

Glutamine is an essential brain metabolite, and dysfunction of synthesis, transfer, and metabolism of glutamine is implicated in numerous neuropathological conditions (Andersen and Schousboe 2023b) (Figure 4). A prominent example of reduced glutamine synthesis is Alzheimer's disease, in which both decreased expression and hampered activity of GS are commonly observed (Olabarria et al. 2011; Jones et al. 2017; Fan et al. 2018, 2021). Reductions in glutamine synthesis arise during the very early phases of disease progression in the 3xTG mouse model of Alzheimer's disease (Kulijewicz‐Nawrot et al. 2013), suggesting that GS dysfunction is a critical early pathological feature. In the 5xFAD mouse model of Alzheimer's disease it was further demonstrated that reduced synthesis of glutamine in astrocytes leads to a direct impairment of neuronal GABA synthesis (Andersen, Christensen, et al. 2021). Intriguingly, a subset of glutamatergic neurons become hyperactive during early Alzheimer's disease progression (Busche et al. 2008, 2012), which may lead to seizures and thereby further accelerate pathology (Vossel et al. 2013, 2016). Taken together, these observations indicate that faulty astrocyte glutamine support disrupts the excitatory–inhibitory balance (Figure 4) mediating synaptic dysfunction in Alzheimer's disease.

Severe reductions in hippocampal GS expression are also observed in patients with temporal lobe epilepsy (Eid et al. 2004; van der Hel et al. 2005). As a major fraction of glutamate recovered from the synapse is converted into glutamine in astrocytes (Figure 1), it has been hypothesized that diminished GS activity will lead to extensive intracellular astrocyte glutamate accumulation, which in turn reduces synaptic glutamate uptake, causing excessive neuronal excitability and seizures (Eid et al. 2004). This hypothesis aligns well with several observations on pharmacological GS inhibition, which causes intracellular glutamate build‐up in astrocytes (Laake et al. 1995), reduces astrocytic glutamate uptake (Zou et al. 2010) and leads to seizures (Eid et al. 2008; Dhaher et al. 2015). In line with this, genetic deletion of GS reduces GLT‐1 and GLAST expression in mice (Zhou, Dhaher, et al. 2019). Another mechanism by which impaired glutamine synthesis could generate seizures is by insufficient support of neuronal GABA synthesis (as hypothesized for Alzheimer's disease above), leading to a reduced inhibitory tone and neuronal hyperexcitation. Interestingly, oral glutamine supplementation has not proved beneficial in experimental epilepsy models but rather exacerbated seizures (Dhaher et al. 2022). This may be explained by the fact that glutamatergic neurons constitute the vast majority of the neuronal population (Braitenberg and Schüz 1998), hence general elevation of brain glutamine levels will not only support GABAergic neurons but even more so the dominant population of excitatory glutamatergic neurons (Figure 1).

Reduced capacity of cellular glutamine transfer has been reported in Huntington's disease. In particular, a lower expression of SNAT3, being a primary astrocytic glutamine transporter, was found in several regions of the R6/2 mouse model of Huntington's disease (Skotte et al. 2018; Hosp et al. 2017). Hampered glutamine efflux from astrocytes may indeed underlie the significant cerebral glutamine accumulation observed in Huntington's disease (Jenkins et al. 2000; Behrens et al. 2002; Andersen et al. 2019; Pépin et al. 2016). Furthermore, impaired glutamine transfer reduces neuronal GABA synthesis in striatal slices of R6/2 mice (Skotte et al. 2018), which may be pivotal as GABAergic medium spiny neurons of the striatum are highly vulnerable to Huntington's disease pathology (Bates et al. 2015). Apart from disrupting glutamate and GABA synthesis, insufficient astrocyte glutamine provision may also have dire metabolic consequences for neurons. Glutamine may act as an anaplerotic substrate in neurons, entering the TCA cycle as α‐ketoglutarate (Figure 3), thus being able to replenish the levels of TCA cycle intermediates. When neurons are deprived of external glutamine support, a metabolic compensation may occur, which increases the capacity for neuronal glutamine oxidation. Such elevated neuronal glutamine metabolism has been demonstrated in models of Alzheimer's disease (Andersen, Christensen, et al. 2021), SSADH deficiency (Andersen et al. 2024) and frontotemporal dementia type 3 (Aldana et al. 2020), further underlining the general importance of glutamine as a metabolic substrate in neurons.

Glutamine synthesis is also the primary route of brain ammonia fixation (Felipo and Butterworth 2002) (Figure 3). As increased ammonia levels directly stimulate glutamine synthesis, astrocytes are the principal regulators of brain ammonia homeostasis (Cooper 2012; Suárez et al. 2002). Sufficient glutamine synthesis capacity becomes crucial during hyperammonemic conditions, for example, hepatic encephalopathy (Häussinger et al. 2022; Butterworth 2002), leading to a massive stimulation of astrocyte glutamine synthesis, which in turn may cause osmotic stress and mitochondrial dysfunction (Norenberg et al. 1991; Albrecht and Norenberg 2006). It has been hypothesized that the elevated glutamine levels during hepatic encephalopathy may facilitate excessive neuronal GABA synthesis, thereby increasing the GABAergic tone and perturbing brain energy metabolism (Sørensen et al. 2022). Elevated brain ammonia levels have also been reported in Alzheimer's disease and Huntington's disease (Seiler 2002; Chiang et al. 2007). The mechanisms and consequences of the heightened ammonia levels in these brain diseases remain to be elucidated. It may be speculated that the aberrant ammonia homeostasis is linked to the disrupted astrocyte glutamine homeostasis as outlined above, but further studies are needed to clarify this.

### 4.2 Impaired Uptake and Metabolism of Glutamate and GABA Are Common Pathological Features

Efficient removal of synaptic glutamate and GABA is paramount to sustain rapid signal transmission with high fidelity. Malfunctioning cellular uptake of glutamate and GABA may lead to great signal imbalances (Figure 4) and is a hallmark of numerous brain diseases. Several studies have reported drastic reductions in brain GLT‐1 expression in Alzheimer's disease (Jacob et al. 2007; Abdul et al. 2009; Hoshi et al. 2018) and Huntington's disease (Liévens et al. 2001; Behrens et al. 2002; Estrada‐Sánchez et al. 2009). Impaired synaptic glutamate clearance may lead to harmful postsynaptic overexcitation, known as excitotoxicity, causing cellular damage and neuronal death (Lewerenz and Maher 2015). Excitotoxicity is a commonly accepted mechanism of neurodegeneration in many cerebral diseases (Lipton and Rosenberg 1994; Sheldon and Robinson 2007). This notion is supported by the immensely deleterious effects of glutamate transporter gene deletion in mice (Tanaka et al. 1997; Rothstein et al. 1996; Petr et al. 2015).

Reducing GLT‐1 expression aggravates disease severity in rodent models of amyotrophic lateral sclerosis (Pardo et al. 2006) and Alzheimer's disease (Mookherjee et al. 2011). Conversely, both pharmacological and genetic inductions of GLT‐1 expression alleviate the pathological progression in mouse models of amyotrophic lateral sclerosis (Guo et al. 2003; Rothstein et al. 2005), Alzheimer's disease (Zumkehr et al. 2015; Hefendehl et al. 2016; Takahashi et al. 2015; Brymer et al. 2023) and Huntington's disease (Miller et al. 2008). Collectively, these studies strongly support a role of disrupted synaptic glutamate clearance as a common mechanism in neurological diseases. A highly interesting observation was made by Fan et al. (2018, 2021), showing that increasing GLT‐1 expression in a mouse model of Alzheimer's disease not only improved glutamate uptake but also restored diminished synthesis, transfer, and metabolism of glutamine (Fan et al. 2018, 2021). These observations align well with studies showing elevated capacity for glutamine synthesis and transport in cultured astrocytes when co‐cultured with neurons or being exposed to exogenous glutamate (Mearow et al. 1990; Mamczur et al. 2015; Fonseca et al. 2005; Tiburcio‐Félix et al. 2018; Gegelashvili et al. 2006). Collectively, these studies serve as an excellent example of how the individual components of the glutamate/GABA‐glutamine cycle are interconnected (Figure 2).

GABA transport is also affected in Alzheimer's disease, as expression of GAT1 and GAT3 is reduced (Fuhrer et al. 2017; Salcedo et al. 2021), coinciding with lower synaptic GABA uptake (Hardy et al. 1987). In addition, several human mutations in GAT1 have been identified, which may lead to epilepsy and autism (Goodspeed et al. 2020; Mermer et al. 2021), whereas GAT3 dysfunction in the amygdala may underlie alcoholism (Augier et al. 2018). As previously mentioned, thalamic GAT expression is restricted to astrocytes (de Biasi et al. 1998), and dysfunction of GAT1 in this region is associated with seizures (Mermer et al. 2021; Pirttimaki et al. 2013).

Altered cellular metabolism of glutamate and GABA may also contribute to disrupted pathological neurotransmitter recycling (Figure 4). Reduced AAT and GDH expression has been reported in Alzheimer's disease (Ciavardelli et al. 2010; Savas et al. 2017; Neuner et al. 2017; Li et al. 2020; Mahajan et al. 2020), suggesting a diminished capacity for glutamate oxidation. However, functional metabolic studies in the 5xFAD mouse model of Alzheimer's disease revealed maintained oxidative glutamate metabolism at several pathological stages (Andersen, Skotte, et al. 2021), which may suggest a functional metabolic compensation. The lower expression of AAT could also have negative effects on the malate–aspartate shuttle (MAS) (McKenna et al. 2006), being essential for transferring glycolytic reducing equivalents from the cytosol into the mitochondrial matrix, needed to sustain energy metabolism. Whether dysfunctional MAS activity contributes to the severe decline in brain energy metabolism in Alzheimer's disease remains to be established. Functional studies in the R6/2 mouse model of Huntington's disease showed a reduced capacity for glutamate oxidation (Skotte et al. 2018). This contrasts with reports of elevated GDH expression and activity in Huntington's disease (Oláh et al. 2008; Zabel et al. 2009) and the lower glutamate metabolism may reflect reduced glutamate uptake or impaired general metabolic capacity of astrocytes.

In contrast to the sustained glutamate metabolism, oxidative GABA metabolism was severely reduced in the 5xFAD mouse (Andersen, Christensen, et al. 2021) and in iPSC‐derived astrocytes of Alzheimer's disease patients (Salcedo et al. 2021), aligning well with lower activity of GABA‐T in brain samples of Alzheimer's disease (Sherif et al. 1992). Intriguingly, a subset of astrocytes may accumulate GABA in Alzheimer's disease offsetting inhibitory signaling (Jo et al. 2014; Wu et al. 2014), which has been attributed to several different mechanisms of pathological astrocyte GABA synthesis (Jo et al. 2014; Wu et al. 2014; Fuhrer et al. 2017; Mitew et al. 2013). However, faulty GABA oxidation may lead to severe GABA accumulation, as observed in SSADH deficiency (Andersen et al. 2024), which could contribute to astrocytic GABA build‐up and disrupted synaptic function in Alzheimer's disease. In relation to brain disease, the GABAergic system has received much less scientific attention than the glutamatergic system. Nonetheless, the relatively small population of GABAergic neurons (Hornung and De Tribolet 1994) is essential in preventing neuronal hyperactivity and excitotoxicity. More functional studies of the GABAergic system in relation to neurotransmitter recycling and brain disease are therefore highly warranted.

### 4.3 Metabolic Dysfunction of Astrocytes Disrupts Lipid Homeostasis and Neurotransmitter Recycling

Astrocyte energy metabolism and mitochondrial function are at the center of the glutamate/GABA‐glutamine cycle (Figure 1). Malfunction of astrocyte metabolism may thereby greatly disturb neurotransmitter recycling and lead to synaptic dysfunction (Figure 4). Astrocytes react strongly to disease or injury, which leads to transient adaptations, including remodeling of physical, morphological, and metabolic functions (Escartin et al. 2021; Sofroniew 2020; Xiong et al. 2022). These adaptive responses may become permanent upon prolonged disease, which is associated with a loss of astrocyte function and accelerated pathological development (Parpura et al. 2012; Verkhratsky et al. 2022). How these adaptations functionally affect astrocyte metabolism is not yet well understood. An elevated oxidative metabolism of astrocytes has been reported during the early stages of Alzheimer's disease (Duong et al. 2021) and in iPSC‐derived astrocytes of Alzheimer's disease patients (Oksanen et al. 2017; Ryu et al. 2021; Salcedo et al. 2024). This aligns well with increased astrocyte glycolytic activity, mitochondrial capacity, and glutamine synthesis in response to an acute inflammatory challenge (Kabiraj et al. 2022; Radford‐Smith et al. 2024). However, during prolonged pathology, the metabolic function of astrocytes generally declines, including mitochondrial dysfunction (Andersen et al. 2022; Gollihue and Norris 2020), which in turn can lead to multiple deleterious effects on the glutamate/GABA‐glutamine cycle.

Brain fatty acid metabolism is gaining scientific attention. It has been argued that fatty acids are poor neuronal fuels, as fatty acid oxidation is associated with elevated production of harmful reactive oxygen species (ROS) and a slow rate of energy production, being incompatible with neuronal metabolism and function (Schönfeld and Reiser 2013). In contrast, astrocytes readily metabolize fatty acids, which provides these cells with a high metabolic versatility, enabling flexible cellular adaptations depending on the metabolic situation (Fernández‐González and Galea 2023). Indeed, human astrocytes are able to elevate fatty acid oxidation when challenged by recurrent low glucose levels in vitro (Weightman Potter et al. 2019). A similar metabolic switch has been observed in astrocytes of a mouse model of Huntington's disease, increasing fatty acid metabolism due to low striatal glucose levels (Polyzos et al. 2019). Astrocytes may utilize fatty acids, not only for energy production, but also to support glutamine synthesis (Andersen, Westi, et al. 2021). Indeed, dietary supplementation with short‐chain fatty acids enhances astrocyte glutamine synthesis and neurotransmitter recycling, and further protects against cognitive impairment, in the APP/PS1 mouse model of Alzheimer's disease (Sun et al. 2023). As mentioned previously, disrupted astrocyte mitochondrial function causes severe lipid accumulation in the brain (Mi et al. 2023). In addition to the cerebral lipid build‐up, impaired mitochondrial function of astrocytes leads to several features mimicking Alzheimer's disease, including synaptic loss, neuroinflammation and cognitive impairment (Mi et al. 2023). This aligns well with the observation that genetic disruptions of astrocyte fatty acid metabolism impairs both working and long‐term spatial memory (Morant‐Ferrando et al. 2023) and that astrocytes display general metabolic impairments during Alzheimer's disease (Andersen et al. 2022).

Lipids are also being exchanged between neurons and astrocytes, which is essential to avoid lipid‐mediated toxicity (Yoon et al. 2021). Transcellular lipid transport is mediated by apolipoprotein E (APOE), which is a critical protein for both the transport and metabolism of lipids (Fernández‐Calle et al. 2022). During intense neuronal signaling, neurons may transfer APOE particles loaded with fatty acids to astrocytes for subsequent metabolism (Ioannou et al. 2019). In *Drosophila*, it has been demonstrated that mitochondrial dysfunction in neurons leads to elevated ROS levels promoting glial lipid accumulation and neurodegeneration (Liu et al. 2015; Byrns et al. 2024). Critically, the expression of a specific APOE variant, APOE4, is the single largest genetic risk factor for the development of Alzheimer's disease (Strittmatter et al. 1993; Saunders et al. 1993) and APOE4 homozygosity is now considered as a distinct form of genetic Alzheimer's disease (Fortea et al. 2024). The APOE4 variant disrupts the shuttling of lipids between astrocytes and neurons (Lin et al. 2018; Qi et al. 2021) and reduces astrocyte uptake and metabolism of lipids (Farmer et al. 2021; Qi et al. 2021; Tcw et al. 2022). APOE4 furthermore impairs astrocyte glucose metabolism, mitochondrial function, and glutamate uptake capacity (Farmer et al. 2021; Williams et al. 2020; Lee et al. 2023; de Leeuw et al. 2022). This, in turn, leads to abnormal lipid accumulation in astrocytes (Farmer et al. 2019; Sienski et al. 2021; Windham et al. 2024), which recently has been associated with neuronal hyperactivity and epileptic seizures (Chen et al. 2023).

APOE production is also closely linked to the mitochondrial function of astrocytes, illustrated by greatly increased APOE levels during the disruption of astrocytic mitochondria (Wynne et al. 2023). Finally, it was recently demonstrated that relieving the APOE4‐mediated lipid burden in astrocytes reduced neurodegeneration in a model of tauopathy (Litvinchuk et al. 2024). It must be noted that, although most APOE is produced and secreted from astrocytes in the brain, APOE also plays functional roles in neurons, microglia, and oligodendrocytes (Blumenfeld et al. 2024). In this regard, microglia may also accumulate lipid droplets (Marschallinger et al. 2020) and APOE4 leads to harmful microglial lipid drop build‐up in Alzheimer's disease (Haney et al. 2024). Nonetheless, the studies above clearly underline that mitochondrial function, particularly of astrocytes, is directly linked to brain lipid homeostasis, which may prove to be a major component of Alzheimer's disease pathology. How brain lipid metabolism is linked to the glutamate/GABA‐glutamine cycle function remains to be fully established. The metabolic function of astrocytes must be explored further in a pathological context as it holds great therapeutic potential (Lee et al. 2022; Verkhratsky et al. 2023).

## 5 Conclusions and Future Perspectives

The glutamate/GABA‐glutamine cycle is essential to maintain synaptic signaling and brain function. The cycle is complex and integrates several cellular processes, including release, uptake, synthesis, and metabolism of glutamate, GABA, and glutamine. All of these processes are interdependent, function in concert, and collectively constitute a highly intricate transcellular system. Our knowledge about the functionality of neurotransmitter recycling has been greatly expanded over the last decades; however, much remains to be uncovered, particularly in connection to brain disease. In this regard, it must always be kept in mind that perturbations of the glutamate/GABA‐glutamine cycle may lead to different functional consequences depending on the affected brain region. Both neurons and astrocytes display distinct region‐specific traits (Kampmann 2024; Zimmer et al. 2024; Brandebura et al. 2023), which may drive the selective regional vulnerability of neurodegenerative diseases. Hence, it is critical to explore neurotransmitter recycling on a region‐specific level in both health and disease. In addition, the seemingly well‐defined interplay between neurons and astrocytes (Figure 1) is currently being expanded to include both oligodendrocytes and microglia. Future studies exploring how these essential glial cells may contribute to or modulate neurotransmitter recycling are strongly encouraged. As all components of the glutamate/GABA‐glutamine cycle are deeply intertwined, it may prove difficult to determine initial causative dysfunctions. This underlines the importance of investigating multiple aspects of neurotransmitter recycling when seeking out potential pathological mechanisms. Many facets of the glutamate/GABA‐glutamine cycle, in particular its intimate connection to cellular metabolism, remain to be fully understood (Rae et al. 2024). Viewing the glutamate/GABA‐glutamine cycle as an integrated system being linked to several central brain processes (Figure 2), instead of individual, yet connected reactions, is needed to facilitate a deeper understanding of how neurotransmitter recycling modulates brain function in health and disease.

## Author Contributions

**Jens V. Andersen:** conceptualization, writing – original draft, visualization, writing – review and editing.

## Conflicts of Interest

The author declares no conflicts of interest.

### Peer Review

The peer review history for this article is available at https://www.webofscience.com/api/gateway/wos/peer‐review/10.1111/jnc.70029.

## Data Availability

The author has nothing to report.
